# Supplementary material for: Content-rich biological network constructed by mining PubMed abstracts
Source: BMC Bioinformatics. 2004 Oct 8;5:147. doi: 10.1186/1471-2105-5-147 (PMC528731; doi:10.1186/1471-2105-5-147)
Supplement: Additional File 5 — The original Chilibot query results of the term "long-term potentiation (LTP)" and 22 other terms, limiting the latest references analyzed to the years 1990, 1995, 2000, and 2004. [file 1471-2105-5-147-S5.bz2 › chilibotAdditionalFile5/ltp2000/html/NMDA.html]

 


**NMDA** (Input: NMDA ) 

---


**Google Searches:** Entire Web  | EDU domain only  | PDF files only

.

**External Links:** OMIM |
LocusLink
| Swissprot
| GeneCards

  
**Maps of NMDA**

Simple
Complete
graph in
radiant
tree
square
layout.

**New Hypothesis !**

**Synonyms** 

- nmda   [PubMed]

**Synopsis**

- Taken together, these data suggest that D1  J Neurosci, 2000 
  [23]

- These results suggest that the increase of calcium influx through **NMDA** channels and L type VOCCs in turn triggering a PKC dependent signaling cascade is a possible cellular basis underlying this seizure like activity induced inhibition of LTP.  J Neurophysiol, 2000 
  [22]

- These results suggest that glutamate can activate CaM kinase II through **NMDA** receptors in the induction of LTP and in turn stimulates the phosphorylation of target proteins such as MAP2 and synapsin I.  Nippon Yakurigaku Zasshi, 1993 
  [22]

- In the mammalian brain, the **NMDA** subclass of glutamate receptors plays a central role in the induction of several forms of use dependent plasticity.  Cell Mol Life Sci, 2000 
  [21]

- Our results suggest that tyrosine phosphorylation of as yet unidentified proteins is responsible for a novel activity dependent depression of **NMDA** receptor function that inhibits synaptic plasticity.  Neuropharmacology, 2000 
  [20]

- Because we have previously shown that acute ethanol can inhibit **NMDA** and AMPA,   Alcohol Clin Exp Res, 1999 
  [19]

- Here we show that, in the CA1 region of the hippocampus, reduction of postsynaptic calcium influx by partial blockade of **NMDA** N methyl D aspartate receptors results in a conversion of LTP to LTD and a loss of input specificity normally associated with LTP, with LTD appearing at heterosynaptic inputs.  Nature, 2000 
  [17]

- Therole of the **NMDA** receptor NMDAR in long term potentiation  [LTP]  LTP is now well established.  J Neurosci Res, 2000 
  [16]

- In fact, at the perforant path granule cell synapse of rats **NMDA** receptor mediated responses are reduced, suggesting a possible change in the threshold for LTP induction at this synapse.  Neurobiol AgingNeurobiol Aging, 2000 
  [16]

- Since an overlap in the behavioral effects of NOS inhibitors and phencyclidine PCP like N methyl D aspartate **NMDA** antagonists has been observed previously, we compared our results with NOS inhibitors to those obtained with PCP.  Behav Pharmacol, 2000 
  [13]

- In human subjects who show a morphologically intact hippocampus that is NOT the primary seizure focus, **NMDA** receptor dependent long term potentiation  [LTP]  LTP and forskolin induced long lasting potentiation are readily induced at the perforant path dentate gyrus synapse.  J Neurosci, 2000 
  [13]

- These results further indicate that mGlu receptor mediated potentiation of **NMDA** responses is NOT essential for the induction of LTP.  Br J Pharmacol, 2000 
  [13]

- Docalcium channels share **NMDA** receptors in plasticity of synaptic transmission in the rat visual cortex?.  Neuroreport, 2000 
  [12]

- Under conditions where the released glutamate can activate **NMDA** receptors, long term potentiation  [LTP]  LTP of the excitatory inputs is induced.  Neuron, 2000 
  [12]

- This fits with the requirement of NO for transmitter release in PC12 cells and **NMDA** treated synaptosomes.  Mol Med, 1996 
  [10]
